# Evaluation of Variability in Gait Styles Used by Dogs Completing Weave Poles in Agility Competition and Its Effect on Completion of the Obstacle

**DOI:** 10.3389/fvets.2021.761493

**Published:** 2021-11-17

**Authors:** Logan D. Eicher, Arielle Pechette Markley, Abigail Shoben, Annika E. Sundby, Nina R. Kieves

**Affiliations:** ^1^Department of Veterinary Clinical Sciences, College of Veterinary Medicine, The Ohio State University, Columbus, OH, United States; ^2^Veterinary Medical Center, College of Veterinary Medicine, The Ohio State University, Columbus, OH, United States; ^3^Division of Biostatistics, College of Public Health, The Ohio State University, Columbus, OH, United States

**Keywords:** agility, weave pole, gait, gait style, canine

## Abstract

**Objective:** The aim of this study was to evaluate and define paw placement patterns for canines completing the weave pole obstacle during canine agility trials. The secondary objectives were to determine the most efficient running style and completion percentages and provide a basis for future studies to evaluate the long-term implications of variants in weave style and predisposition to injury. We hypothesized that dogs would display definitive gait patterns and that a single stepping pattern would yield faster run times compared to double stepping patterns.

**Animals:** A total of 1,377 video recordings of dogs completing weaves poles at the American Kennel Club 2019 National Agility Championship were viewed.

**Procedures:** Competition videos were reviewed as dogs attempted completion of the weave pole obstacle. Data collected included front limb and rear limb paw placement styles, time to complete the obstacle, and demographic data.

**Results:** Attempts could be classified into one of five styles based on front and rear paw placement, with no one style dominant. Weave style differed by height and breed, with taller dogs and Border Collies preferring a single stepping style. Weave times were significantly faster for competitors using a single stepping style vs. other gaits.

**Conclusions and Clinical Relevance:** This study found five identifiable gait styles used by dogs running weave pole obstacles, with front feet single stepping yielding significantly faster run times compared to other gaits. A clear classification of running styles will allow future studies to evaluate different stresses on joints, such as the shoulder, between varying gait styles, which could lead to recommendations for injury prevention.

## Introduction

Agility has become increasingly popular in the world of canine performance with over 1.2 million entries into American Kennel Club (AKC) events alone in 2019 (personal communication, Carrie DeYoung, Director of AKC Agility, June 30, 2020). The timed event involves handlers directing a canine competitor through a pre-set course of obstacles (between 14 and 20) that may include an A-frame, dog walk, seesaw, pause table, open tunnel, weave poles, and bar jumps within a set time frame (1, 2). The sport is physically demanding, with reports of 32–42% of dogs experiencing at least one injury while involved in the sport (3–6). There is also evidence to support that, following orthopedic injury, only 67% of dogs were able to return to competition and that jump height had to be decreased in 47% of those dogs that subsequently returned to competition (7). Currently, the sport lacks data to support a clear definition of running styles, the most efficient patterns for all obstacles, and the long-term implications for injury risk and longevity of the competitor based on their running style. In order to make evidence-based recommendations for training in agility and design of agility courses, studies to better understand running patterns and their relation to injury must be performed.

Normal canine gait patterns are well-described and include the walk, amble, pace, trot, canter, and gallop. Gait can be affected by surface properties and whether an animal is moving in a straight line or a curvilinear path (8). The agility weave pole obstacle is unique in that it requires the dog to move not only forward at a chosen pace but also in a lateral plane from side to side. During this obstacle, the dog must navigate a series of six to 12 upright poles 40 in. in height and set at 24 in. apart (2). The dogs must always enter the obstacle with the first pole to their left and not skip the subsequent poles. The number of poles varies with the level of agility competition. A study by Siniscalchi et al. evaluated canine laterality in relation to paw preference during the performance of weave poles and the A-frame (9). This study found that dogs performed better when their handlers were positioned in their right visual hemifield. However, to date, no description of different gait patterns nor any assessment of variation in success or speed with different gait styles used, while completing the weave pole obstacle, has been made.

Therefore, the aim of this study was to evaluate and clearly define the gait pattern and paw placement used by canine athletes completing weave poles during a competition. Secondarily, we aimed to determine the most efficient running style, including successful completion percentages between gaits, and to provide a basis for future studies to evaluate the long-term implications of variants in weave style and predisposition to injury. We hypothesized that clear definitive gait styles would be identified and that a gait using only a single forelimb compared to both forelimbs would yield significantly faster run times through the weave poles but have a lower successful completion rate of the obstacle.

## Materials and Methods

Video recordings from the AKC 2019 National Agility Championship were reviewed as dogs attempted the completion of weave poles during both standard (STD) and jumpers with weaves (JWW) competition runs. The videos were created by 4LeggedFlix for publicly available live viewing of the event. Prior to data collection, four reviewers (NRK, AS, APM, AES) reviewed a small number of the available videos and identified specific paw placement patterns for front and rear limbs. Then, all video was reviewed in YouTube, utilizing slow motion (0.25 × speed) by a single observer (LDE). Time was recorded using the time stamp data in YouTube or that which was provided by 4LeggedFlix on their videos.

Weaves were the sixth obstacle in STD, with a 180° approach with the handler to the right of the dog. Camera position was nearly straight on to the weaves, and dogs were weaving toward the camera. Weaves were the third to the last (18th) obstacle in JWW and were on the path of the dog following a curve to the right (with the handler to the left of the dog). In JWW, the camera was again nearly straight to the weaves, but the dogs were weaving away from the camera. On both courses, both the previous and the next obstacle were a common single bar jump. The courses and camera position were very similar across all height classes, although due to the large nature of the event, dogs from different heights competed in different rings, so minor variations may have existed. The surface in all rings was groomed and packed with dirt.

Specific data collected on each run included whether or not the weaves were successfully completed on the first attempt, front limb and rear limb paw placement style, and time to complete the obstacle. The time of completion was defined as the time when the shoulder of the dog passed the first weave pole to the time when their entire body cleared the last weave pole. Primary paw placement style was recorded for the successful weave attempt if available or for the first failed attempt (if possible to observe) on runs where the dog never successfully completed the obstacle. Additional information recorded for each run included the breed of the dog, competition class (STD or JWW), and the standard measured height class of the dog (16, 20, or 24 in.) as a categorization of the height of the dog at the withers. Dogs in the 16-in. class measured between 14 and 18 in. at the withers, the 20-in. dogs measured between 18 and 22 in., and the 24-in. dogs were taller than 22 in.

Due to the nature of the event observed, the same dog could be observed twice (once for STD and once for JWW). As such, the descriptive statistics and reported distribution of paw placement styles are reported separately for STD and JWW classes. Differences in the distribution of style by height and by breed (Border Collie vs. others) were evaluated using chi-square tests. Due to the small number of faulted runs, Fisher's exact test was used to assess differences in the percentage of clean first weave attempts by style. Differences in mean weave times by style were examined using generalized estimating equations with robust standard errors to account for both correlated observations (same dog in both STD and JWW) and possible heteroscedasticity. When comparing pairwise means between weave styles, the Holm correction was used to control the overall type I error rate. Statistical significance was set at *p* < 0.05, and analyses were conducted using Stata (StataCorp, College Station, TX, USA).

## Results

A total of 1,377 weave attempts were reviewed. Weave style could be determined for 1,364 of the attempts (99%). Weave style could not be determined for the animals that failed to complete more than two poles of the obstacle. The most common dog breed represented was the Border Collie comprising 38% (530) of all attempts, followed by a wide distribution of other dog breeds. A total of 53 unique breeds were observed (Table 1). Full signalment (sex and age) of each competitor was not publicly available and therefore could not be reported here.

**Table 1:** Distribution of all dog breeds included in the current study.

**Dog breed**	***N***	**%**
Border collie	530	38.49
Golden retriever	140	10.17
Shetland sheepdog (Sheltie)	107	7.77
Australian shepherd	102	7.41
Mixed breed/all-american	88	6.39
Labrador retriever	74	5.37
Poodle (standard)	26	1.89
Vizsla	25	1.82
Miniature american shepherd	21	1.53
Belgian malinois	18	1.31
Doberman pinscher	18	1.31
Nova scotia duck tolling retriever	17	1.23
Brittany	16	1.16
German shepherd dog	16	1.16
Belgian tervuren	13	0.94
Portuguese water dog	13	0.94
Dalmatian	11	0.8
Flat coated retriever	9	0.65
Pumi	9	0.65
Australian sattle dog	8	0.58
Belgian sheepdog	8	0.58
Cocker spaniel-english	7	0.51
Rottweiler	7	0.51
Weimaraner	7	0.51
Boxer	6	0.44
Collie (rough and smooth)	6	0.44
English springer spaniel	6	0.44
Rat terrier	6	0.44
Whippet	6	0.44
Giant schnauzer	5	0.36
English setter	4	0.29
German shorthaired pointer	4	0.29
Irish setter	4	0.29
Manchester terrier (standard and toy)	3	0.22
Siberian husky	3	0.22
American eskimo	2	0.15
Bearded collie	2	0.15
Briard	2	0.15
Chesapeake bay retriever	2	0.15
Chinese crested	2	0.15
Curly coated retriever	2	0.15
Irish water spaniel	2	0.15
Italian greyhound	2	0.15
Keeshond	2	0.15
Kerry blue terrier	2	0.15
Lagotto romagnolo	2	0.15
Norwegian elkhound	2	0.15
Samoyed	2	0.15
Shiba inu	2	0.15
Soft-coated wheaten terrier	2	0.15
Standard schnauzer	2	0.15
Beagle	1	0.07
Cocker spaniel-american	1	0.07

**Figure 1:**
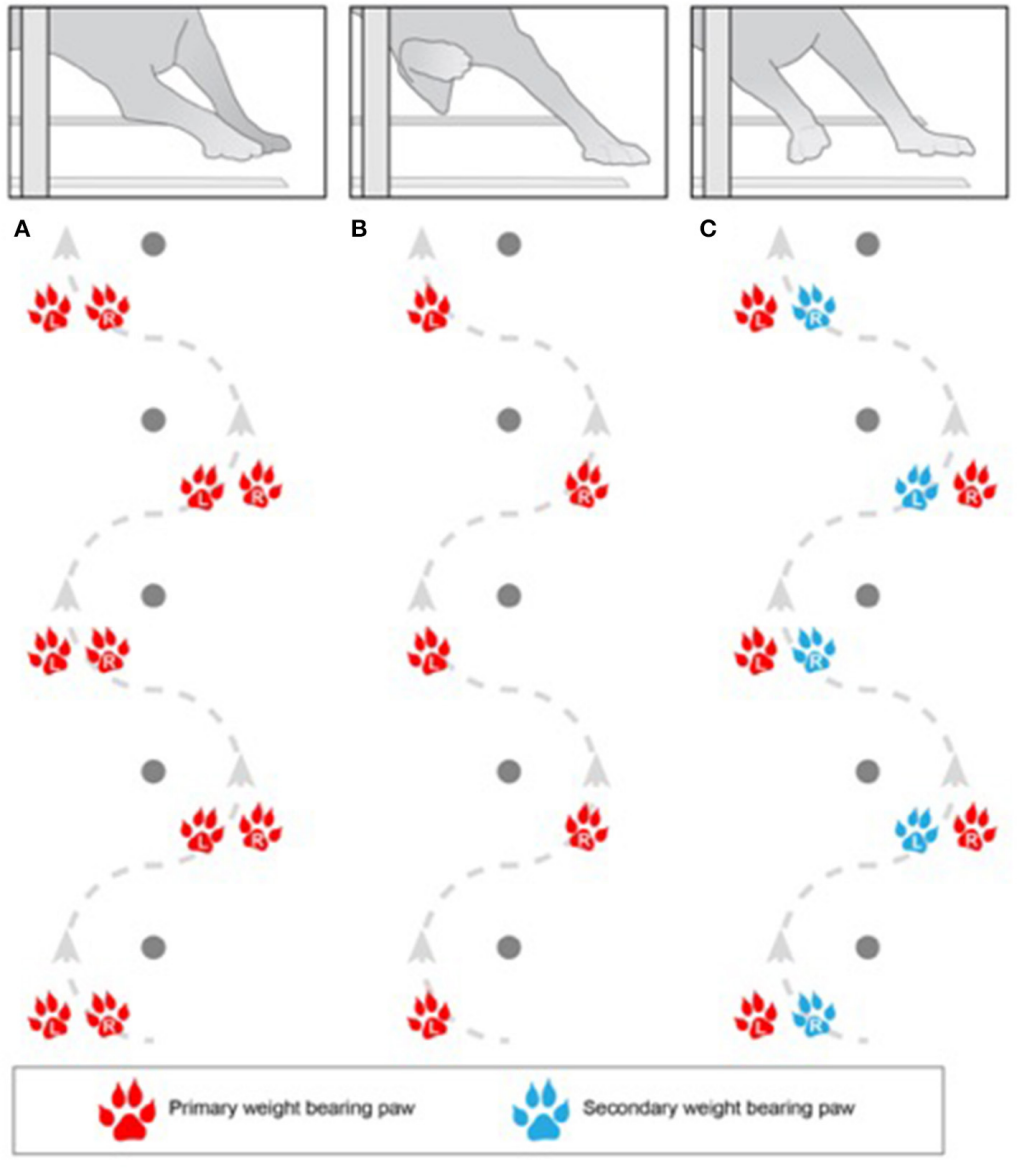
Illustration of front feet paw placement patterns used by canine competitors completing the weave pole obstacle in agility competition. **(A)** Front feet hopping, **(B)** front feet single stepping, and **(C)** front feet double stepping. If no clear paw placement pattern was seen (i.e., the dog used multiple patterns throughout the run) or the competitor walked through the weave poles, these were classified as multiple steps/walking. Dogs were classified as hopping if the competitor used both paws, landing on both at the same time, and used both paws to simultaneously push off between weave poles. Double stepping was assigned when the competitor used both paws but landed on one paw at a time, making initial contact with the lateral paw followed by the medial paw, and then pushed off with both feet between weave poles. This was compared to single stepping when the competitor landed and used only one paw—the dog landed on only the lateral paw and pushing off with only the lateral paw between weave poles. With this gait, the medial paw did not contact the ground between weave poles at any time.

Five separate common gait patterns were observed for dogs when weaving (Table 2 and Figure 1). A front feet hopping (FFH) or front feet double stepping (FFDS) pattern for the front limbs was nearly always accompanied by a rear foot hopping style for the pelvic limbs, so these styles were reported when observed regardless of rear limb paw pattern. In contrast, attempts where the dog used a front feet single stepping (FFSS) style (*n* = 518) were split as to if the dog used a rear foot single stepping (RFSS) style (*n* = 145, 28%) or a rear feet double stepping (RFDS) style (*n* = 373, 72%) approach. Thus, the FFSS style was further categorized as FFSS/RFSS or FFSS/RFDS. The final category of gait pattern observed was when the dog took multiple steps (MS) between the poles or was walking.

**Table 2:** Paw placement patterns for front limbs used by canine competitors completing the weave pole obstacle in agility competition.

**Paw placement**	**Paw placement description**
Multiple steps/walking	No clear paw placement pattern; competitor walks through the weave poles
Hopping	Competitor uses both paws, landing on both at the same time, and uses both paws to simultaneously push off between weave poles
Double stepping	Competitor uses both paws but lands on one paw at a time, making initial contact with the lateral paw followed by the medial paw, and then pushes off with both feet between weave poles
Single stepping	Competitor lands using only one paw, landing on only the lateral paw, and pushes off with only the lateral paw between weave poles. Medial paw does not contact the ground between weave poles at any time

Of all 1,377 weave attempts, the majority (*n* = 1,238, 89.9%) were successfully completed on first attempt. Of the failed first attempts, 91 (65.5%) were at entry into the obstacle. On the STD course, there was no significant difference in percent clean run by weave style (*p* = 0.21). A marginal difference by style was observed on the JWW course (*p* = 0.034), with a higher likelihood of a clean run associated with the FFSS style relative to FFH and FFDS (Table 3).

**Table 3:** Percentage of observed runs clean on first attempt by style and course.

	**Standard**		**Jumpers**	
	***N* (%) (*n* = 695)**	**% clean (*p* = 0.21)**	***N* (%) (*n* = 669)**	**% clean (*p* = 0.034)**
Multiple steps	69 (9.9)	88.4%	46 (6.9)	87.0%
Front feet hopping	153 (22.0)	93.5%	260 (38.9)	82.3%
Front double stepping	199 (28.6)	95.5%	119 (17.8)	84.9%
Front single, rear single	84 (12.1)	96.4%	61 (9.1)	93.4%
Front single, rear double	190 (27.3)	92.1%	183 (27.4)	91.3%

*Percentage clean was not different by style on the standard course (p = 0.21) and was marginally different on the jumpers with weaves course (p = 0.034)*.

There were significant differences in weave style by height and breed. Dogs competing in the 16-in. height class were much more likely to use a FFH style than taller dogs competing in the 20- or 24-in. height class. The taller dogs were more likely to use a FFDS or FFSS approach (*p* < 0.001 for both STD and JWW, Table 4). Holding dog height constant by considering only the 20-in. height class, non-Border Collie breeds were significantly more likely to use FFH or FFDS when compared to the Border Collie who predominantly used a FFSS approach (*p* < 0.001, Table 5).

**Table 4:** Weave style by measured jump height category reported as *N* (%).

	**Standard**			**Jumpers**		
	**16-in. (*n* = 176)**	**20-in. (*n* = 381)**	**24-in. (*n* = 138)**	**16-in. (*n* = 167)**	**20-in. (*n* = 370)**	**24-in. (*n* = 132)**
Multiple steps	14 (8.0)	36 (9.5)	19 (13.8)	7 (4.2)	22 (6.0)	17 (12.9)
Front feet hopping	90 (51.1)	47 (12.3)	16 (11.6)	134 (80.2)	101 (27.3)	25 (18.9)
Front double stepping	39 (22.2)	127 (33.3)	33 (23.9)	4 (2.4)	93 (25.1)	22 (16.7)
Front single, rear single	16 (9.1)	50 (13.1)	18 (13.0)	4 (2.4)	33 (8.9)	24 (18.2)
Front single, rear double	17 (9.7)	121 (31.8)	52 (37.7)	18 (10.8)	121 (32.7)	44 (33.3)

*Significant differences were observed in both standard and jumpers with weaves (p < 0.001 for both)*.

**Table 5:** Weave style by breed among dogs in the 20-in. height category reported as *N* (%).

	**STD**		**JWW**	
	**All other breeds (*n* = 179)**	**Border collies (*n* = 202)**	**All other breeds (*n* = 176)**	**Border collies (*n* = 194)**
Multiple steps	26 (14.5)	10 (5.0)	15 (8.5)	7 (3.6)
Front feet hopping	36 (20.1)	11 (5.5)	65 (36.9)	36 (18.6)
Front double stepping	78 (43.6)	49 (24.3)	59 (33.5)	34 (17.5)
Front single, rear single	1 (0.6)	49 (24.3)	2 (1.1)	31 (16.0)
Front single, rear double	38 (21.2)	83 (41.1)	35 (19.9)	86 (44.3)

*Significant differences were observed in both standard (STD) and jumpers (JWW) (p < 0.001 for both)*.

**Figure 2:**
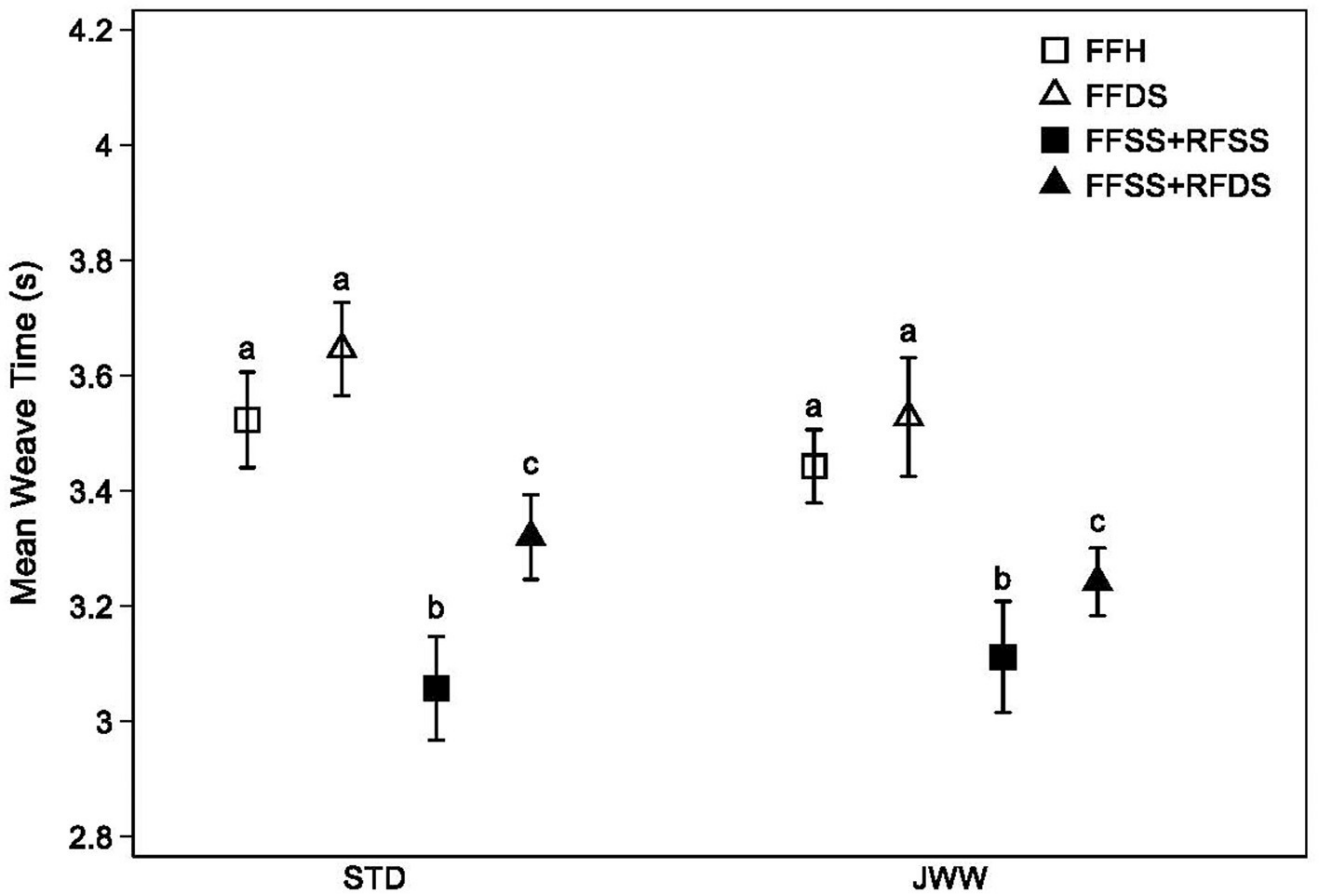
Mean weave time (seconds) between different gaits used while completing the weave pole obstacle. Competitors that used front feet single stepping paired with rear feet single stepping or rear feet double stepping completed the weave poles significantly faster than those that used front feet hopping or front feet double stepping. Error bars indicate 95% confidence intervals for the mean. Different letters indicate means within course that were different from each other, with Holm correction (*p* < 0.05); all pairwise differences were significant except front feet hopping vs. front feet double stepping (unadjusted *p* = 0.037 for standard and *p* = 0.17 for jumpers). STD, standard course; JWW, jumpers with weaves.

Weave times were significantly different based on weave style. As expected, the MS style was associated with dramatically longer mean times (>5 s), where all other styles had means <3.7 s (*p* < 0.001, Figure 2). Of the remaining styles, the FFSS/RFSS style was fastest (*p* < 0.001 for all pairwise comparisons), followed by the FFSS/RFDS style. Both FFSS styles were significantly faster than either FFH or FFDS (*p* < 0.001) and such differences persisted after adjusting for the dog's jump height and breed (Table 6). FFH was marginally faster than FFDS (*p* = 0.004), in unadjusted models, but this difference was the smallest observed and was no longer observed in models adjusting for jump height (Table 6).

**Table 6:** Estimated mean speed differences (and 95% confidence intervals) by weave style from generalized estimating equation regression models.

	**Unadjusted**	**Adjusted for jump height**	**Adjust for jump height and breed^**a**^**
Multiple steps	1.91 (1.68, 2.15)^b^	1.75 (1.53, 1.96)^b^	1.76 (1.55, 1.97)^b^
Front feet hopping	Reference	Reference	Reference
Front double stepping	0.13 (0.04, 0.22)^b^	0.01 (−0.07, 0.10)^c^	0.05 (−0.03, 0.14)^c^
Front single, rear single	−0.39 (−0.49, −0.30)^b^	−0.55 (−0.64, −0.46)^b^	−0.31 (−0.40, −0.21)^b^
Front single, rear double	−0.19 (−0.27, −0.11)^b^	−0.35 (−0.43, −0.27)^b^	−0.20 (−0.29, −0.12)^b^

*^a^ Border Collie vs. non-Border Collie. ^b^Significantly different than all other styles (using Holm's correction for multiple comparisons).*

*^c^Significantly different from all other styles except front feet hopping*.

## Discussion

This study revealed that dogs do show variance in gait style while completing the weave pole obstacle during agility competitions. We were able to define four distinctive gait variations for front and rear limb patterns (Table 2) and show that all dogs could be classified into one of five common combinations of front and rear limb styles. These can be used in future studies to help determine differences in biomechanical forces between styles and correlation with injury type and risk.

In evaluating the completion time of the obstacle, FFSS/RFSS yielded significantly faster run times compared to other gaits. We suspect this to be due to the shorter contact time required when stepping with a single foot, though additional evaluation is needed to confirm this hypothesis. Despite less ground contact time and increased speed of completion, FFSS/RFSS did not have a negative impact on successful completion of the weave poles; thus, we were unable to fully accept our hypothesis. In fact, there was a suggestion that dogs using FFSS/RFSS were more likely to complete the weaves cleanly on the first attempt compared to other gait styles. This may be reflective of a higher level of discipline and acuity of this subset of competitors. The high successful completion percentage and speed associated with FFSS/RFSS is likely skewed by the population of dogs in this study as they were competing at a national event that requires specific qualifications for entry. It is possible that differences in successful completion percentage and speed by weave style are more notable at lower levels of competition.

What remains unknown is the difference in forces placed upon the forelimbs and the difference in kinematics between gait styles. It is possible that one type of gait style places more force on the forelimb, the shoulder joint in particular, that could lead to a predisposition toward injury. Shoulder injuries are the most common injury that occurs in agility dogs (4–6), and such knowledge could change the way dogs are trained for agility and assist in recommendations for the prevention of injury in this population of dogs. This is an area for future prospective kinetic and kinematic studies.

Some initial work has been done as to assessing variation in locomotion during canine gait between breeds. It has been demonstrated that kinetic data varies between breed (10). Additionally, one study assessed differences in kinematics of joints in the pelvic limb of Greyhounds and Labrador Retrievers (11). They found significant differences in the kinematic patterns of all joints assessed. This is likely true of thoracic limb joints between breeds with substantial conformational differences, though to date it has not been assessed. A more recent study also evaluated differences in hind limb kinematics of four breeds (Beagle, French bulldog, Malinois, and Whippet) of varying conformations using biplanar, high-frequency fluoroscopy, and 3D kinematics. They found significant differences in how the breeds moved (12). This study only evaluated forward linear motion on a treadmill. Assessment during more complex motion still needs to be performed.

While the locomotion of canines cannot be compared to bipedal humans, numerous studies have evaluated human gait patterns during running. Variation in foot-strike pattern is prevalent and affects both runner efficiency and injury rates in athletes (13–15). Furthermore, variation in foot-strike is noted between elite and sub-elite athletes (16). Research on equine foot-strikes shows that equine distal limb lameness can often be attributed to variation in hoof contact areas (17), so understanding variations in canine agility obstacle gait preferences may lead to earlier recognition and diagnosis of injuries. Though foot-strike is not equivocal to gait pattern, the variation in patterns seen in this study and the prevalence of the FFSS should be investigated to see if experienced canine athletes prefer one gait pattern over another as compared to novices.

A previous study evaluating limb forces when jumping found a significant difference in limb stiffness when landing between beginner and experienced dogs (18). This increase in stiffness was associated with higher limb compression during stance phase. It was hypothesized that larger eccentric muscle contraction due to the increased compliance that occurred during landing may be associated with injury (18). It is unknown whether there are similar variations in forelimb forces between beginner and experienced dogs as they complete the weave poles. Future studies evaluating differences between gait pattern preference and forces between beginner and experienced dogs may provide crucial information regarding training protocols, injury prevention, and competition readiness. If a dog maintains the same weave gait pattern throughout their career, then changes to the gait pattern of a dog may be indicative of injury development. Further investigation is needed to determine if there is a notable difference in gait patterns and limb forces when comparing novice and experienced dogs.

The differences observed in gait pattern between the height classes is likely correlated to variation in conformation and size of the canine athletes. Previous studies have demonstrated biomechanical variability in locomotion between breeds and sizes of dogs (10, 19–23). Taller dogs in the 20- and 24-in. height class were much less likely to use a FFH technique as compared to the 16-in. height class. This may be due, in part, to their larger body size and ability to propel themselves through the poles due to increased stride length, which may lead to a natural preference for FFSS/FFDS techniques. While the FFH technique was found to have a longer time of completion, attempting to train dogs with shorter natural stride a different technique may be limited by the structural conformation of the competitor, which could ultimately have a negative impact on performance and result in increased injury. This is unknown. Although we did not observe dogs in the 12-in. height class due to observational limitations, we predict that they would preferentially prefer FFH or MS gait patterns due to their short stature and inability to perform the other aforementioned techniques. This requires further evaluation to corroborate. Future studies are needed to evaluate differences in weave gait pattern preference as well as kinetics and kinematics during weave performance between different breeds of dogs.

It is unknown whether the predominance of Border Collie for FFSS, and faster associated run times, is due to their conformation or whether other factors such as confidence, drive, trainability, etc., could influence their natural preference for this style. Even within the Border Collie breed there is variability in conformation, with differences in shoulder angulation, length of back and loin, and rear limb angulation being notable. Variability of conformation has been shown to affect movement within breeds (24). This effect may be even greater when performing highly physical activities such as agility, compared with walking and trotting. It was not obvious, based on this study, that Border Collies are unilaterally faster regardless of weave style or if instead Border Collies are faster because they are more likely to utilize a more efficient weave style. Studies are needed to specifically evaluate how structural conformation, specifically shoulder angulation and body length, may affect weave gait pattern preference, speed, as well as the kinetics and kinematics, and how that is related to musculoskeletal injury development.

There are a number of other variables that may affect weave gait style preference and performance. Overall speed and drive, weave entry and exit angle, obstacle type and placement location before and after the weaves, surface type and maintenance, handler location, handling technique, and handler movement during obstacle performance could all potentially affect the gait pattern, speed, and performance of an individual dog. Other variables, such as initial weave training technique, increased/decreased motivation, and changes in strength and flexibility, could also affect the preference of an individual dog for one gait style vs. another. Additional studies are needed to evaluate these variables and how they influence the weave gait pattern of an individual dog as well as whether they have any influence on the kinetics and kinematics of weave performance.

This study only evaluated one level of competition, a national level, which is a limitation particularly in regard to the completion success rate reported. It is possible that less experienced competitors have a lower completion success rate, and this should be evaluated in future studies. The level of competition was not considered a variable to have affected the description of the gait patterns noted in the present study. Additionally, breed distribution may vary between a national level competition and the general agility population. There may be additional breed differences between gait styles that were not accounted for in this population due to the large percentage of Border Collies competing. Additional limitations include the single, fixed camera angles of the videos assessed, which made a clear interpretation of gait style difficult to evaluate in some runs, most notably in the shorter height classes. However, the majority of runs were able to be assigned a gait pattern. In particular, the fixed-angle cameras made the evaluation of lower height classes nearly impossible. Video analysis via YouTube is also a potential limitation, as YouTube does not allow the fine degree of analysis as with other video programs. However, the time differences observed were so large that they are unlikely to be due to measurement errors in recording.

To the knowledge of the authors, this is the first study to define the paw placement style and gait pattern used by canine competitors completing the agility weave pole obstacle. The clear classification of running styles found in this study will allow future studies to be designed to help evaluate different stresses on joints, particularly the shoulder and elbow, between varying gait styles. This could help assess for predisposition to injury and long-term implications for athletes using a particular running style.

## Data Availability Statement

The raw data supporting the conclusions of this article will be made available by the authors, without undue reservation.

## Author Contributions

LE participated in data collection, data evaluation, and writing the manuscript. AM and NK assisted in study design, data collection, data evaluation, and writing the manuscript. ASh participated in study design, statistical analysis, and writing the manuscript. ASu participated in data collection and editing of the final manuscript prior to submission. All authors contributed to the article and approved the submitted version.

## Conflict of Interest

The authors declare that the research was conducted in the absence of any commercial or financial relationships that could be construed as a potential conflict of interest.

## Publisher's Note

All claims expressed in this article are solely those of the authors and do not necessarily represent those of their affiliated organizations, or those of the publisher, the editors and the reviewers. Any product that may be evaluated in this article, or claim that may be made by its manufacturer, is not guaranteed or endorsed by the publisher.
